# Local Crystallographic Texture of a Nummulite (Foraminifera) Test from the Eocene Deposits of the Crimea Peninsula

**DOI:** 10.3390/biology12121472

**Published:** 2023-11-27

**Authors:** Alexey Pakhnevich, Dmitry Nikolayev, Tatiana Lychagina

**Affiliations:** 1 Borissiak Paleontological Institute, Russian Academy of Sciences, 117647 Moscow, Russia; alvpb@mail.ru; 2 Frank Laboratory of Neutron Physics, Joint Institute for Nuclear Research, 141980 Dubna, Russia; dmitry@nf.jinr.ru

**Keywords:** nummulites, Eocene, local crystallographic texture, pole figures, X-ray diffraction, X-ray microtomography images

## Abstract

**Simple Summary:**

The tests of the unicellular protozoan *Nummulites distans* (Cenozoic era, Paleogene period) were studied using X-ray diffraction. In addition, the images obtained using X-ray microtomography and scanning electron microscopy were analyzed. A high degree of the calcite crystal orientation ordering that the test consists of has been revealed. It is higher than that of calcite from the shells of the bivalve mollusk *Placuna placenta*, whose shell is formed by a special multicellular tissue. So, it is found that already unicellular organisms have strict control over the crystal arrangement.

**Abstract:**

Unicellular protozoa form calcium carbonate tests. It is important to understand the features and mechanisms of its formation. This may shed light on the processes of shell formation in metazoans. One of the most important characteristics of the Protozoa carbonate test is the degree of crystal ordering that can be described by crystallographic texture. The crystallographic texture data of calcite in the foraminifera *Nummulites distans* (Deshayes) test from the Eocene deposits (Cenozoic, Paleogene) of the Crimea Peninsula are obtained using X-ray diffraction. A very strict orientation of the crystals is revealed. The calcite texture sharpness is several times greater than in the shells of the bivalve mollusk *Placuna placenta* (Linnaeus), measured by the same method. It also exceeds the crystallographic texture and sharpness of the same mineral in the shells of the bivalves of *Mytilus galloprovincialis* (Lamarck), studied by neutron diffraction. It is concluded that a high level of control during test formation is already characteristic of protozoa. Studying the processes involved in the formation of a very sharp crystallographic texture can become an important direction for creating nature-like materials with desired properties.

## 1. Introduction

**Figure 1:**
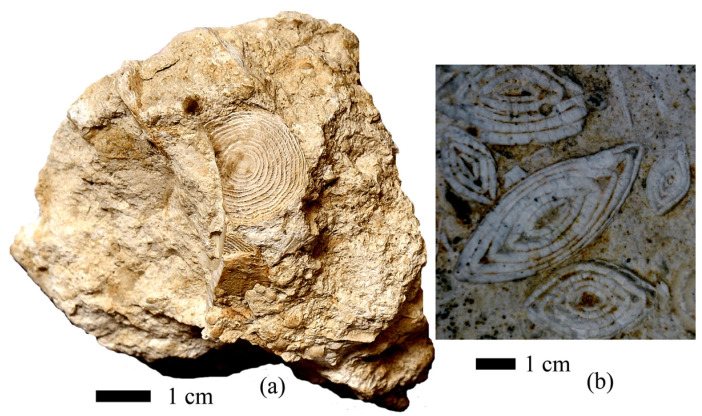
Nummulite tests in the rock: (**a**) specimen no. OF 2562/41, collection of the Kolomna Museum of Local Lore, nummulite test on a chip, Crimea Peninsula, Eocene; (**b**) Nummulite tests on a weathered cleavage, Skalistoye village, Crimea Peninsula, Eocene (photo by M.I. Leonova).

Despite the fact that protozoa are unicellular organisms, they are able to create complex external mineral skeletons. These are multichambered porous foraminiferal tests that are architecturally complex, consisting of crossbars, radiolarian shells, goblet-shaped ciliates, and tintinnids. Their skeletons are composed of a number of minerals, such as calcite, aragonite, opal, amorphous calcium phosphate, celestite, barite, iron oxides [1,2], and organic matter. Foraminifera are able to build secretory and agglutinated tests, holding together elements of the bottom sediment. The cell size of foraminifers can reach several centimeters, and the tests of the largest of them, nummulites, show up to 16 cm [3]. Nummulites have a flattened biconvex lenticular test divided into chambers. The test consists of several spiral whorls (Figure 1).

**Figure 2:**
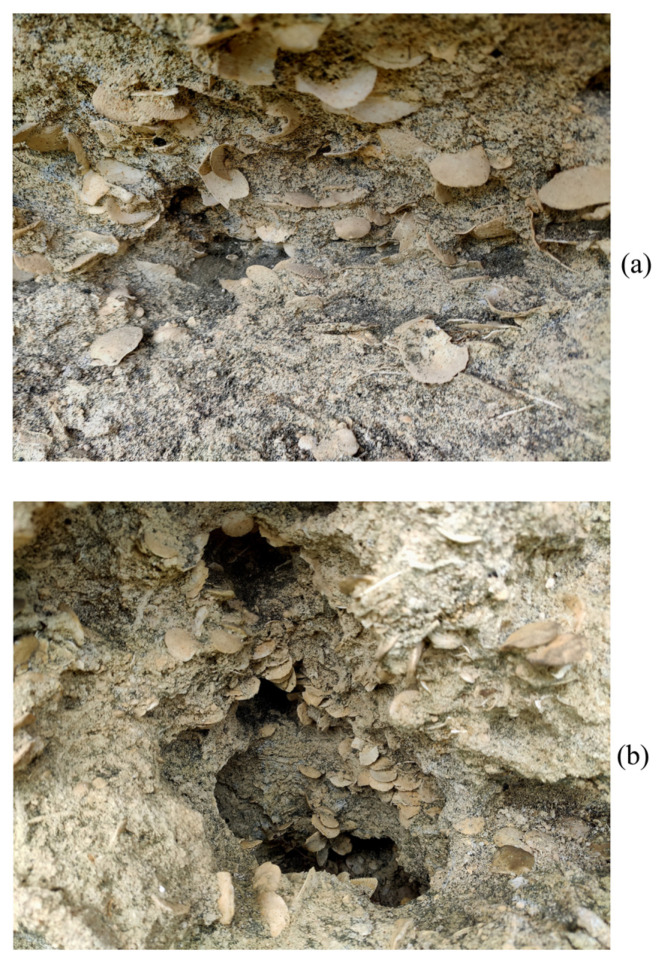
Nummulite limestones, Mount Ak-Kaya, Crimea Peninsula, Eocene (photo by M.I. Leonova): (**a**) Accumulation of nummulite tests in the rock, (**b**) Caverns with water-washed nummulite tests.

Nummulites are characterized by a planispiral growth pattern [4]. It is possible that the symbiosis with algae described by a number of representatives of large foraminifers is important for the growth of such a large test [5]. Tests were performed on calcium carbonate. Nummulites are of stratigraphic importance for the division of the Upper Cretaceous–Oligocene deposits of Europe, Asia, and Africa [6,7,8,9,10,11,12,13,14,15,16]. Nummulite tests are of rock-forming significance (Figure 1 and Figure 2) [17]. Due to the complexity of the structure and the size of the cell, it is absolutely clear that the nummulite test is not a single crystal. But how much one cell can control the ordering of crystals in the test remains an open question.

The ordering of crystals can be described by crystallographic texture, which is a collection of crystalline orientations in a polycrystalline sample. Quantitative information about crystallographic texture is contained in pole figures that are experimentally measured by methods based on X-ray or synchrotron radiation, electron, or thermal neutron diffraction [18,19,20,21,22]. The pole figures are two-dimensional distributions of relative volumes for specific crystallographic directions on a unit sphere. The more ordered the mineral crystals are, the higher the intensity on the pole figure (pole density), expressed in units of isotropic distribution (multiple random distribution, mrd). An increase in pole figure intensity is interpreted as an increase in crystallographic texture. A pole density value equal to unity means that the corresponding crystallographic planes are uniformly distributed in a sample in all directions.

The pole figure measurements have been developed for metals and alloys [22,23]. At present, there is a great interest in the crystallographic texture study of biological samples [24,25,26,27,28,29,30,31,32,33,34,35].

Our goal is to investigate the features of the local crystallographic texture of the nummulite tests (*Nummulites distans* Deshayes, 1838). Previously, the crystallographic texture of nummulite tests was not measured, especially on a natural surface. Only measurements were made of sedimentary rocks containing nummulite [36]. However, there are a number of studies of the calcite crystallographic texture of smaller foraminifera, which, due to their size, were measured using electron backscattered diffraction (EBSD) [37,38,39,40] or with a diffractometer based on a synchrotron source [41]. Using the EBSD method, it was possible to study foraminiferal tests very locally. It was established that the test wall of foraminiferal rotaliids consists of mesocrystals, which, in turn, are formed by mosaic polycrystalline [38]. Interestingly, rotallids also belong to foraminifera with a hyaline microstructure of the test wall [42,43]. The findings on fossil foraminifera are interesting. *Gyroidinoides soldanii* (d’Orbigny, 1826) shows low texture sharpness with almost no preferred orientation. Over millions of years, the size of the crystals and the test wall structure of *G. soldanii* and *Cibicides grimsdalei* (Nuttall, 1930) remained unchanged. In addition, the crystallographic texture remains constant. The EBSD method is relevant for analyzing the crystallographic texture of individual structures in small objects. It is very difficult to use it for comparison, even with the results of measurements using X-ray diffraction, since the area of the analyzed volume differs by tens of times.

It was previously shown that the crystallographic texture of calcite and aragonite in the shells of bivalve mollusks is very stable over time [30,32], in different parts of the habitat under different climatic conditions [34], and under the influence of trace elements [35]. Some objects showed unusually high sharpness of crystallographic texture [30,33]. For example, the bivalves of the family Mitilidaie have a pole density (0006) maxima greater than 10 mrd.

We measured the nummulites crystallographic texture using X-ray diffraction. To investigate the features of texture, we used both the sharpness values of the crystallographic texture (pole density maxima) and the distribution of pole figure isolines. Besides, diffraction patterns, virtual sections, scanning electron, and light microscopy images were analyzed.

## 2. Materials and Methods

The nummulite tests from the Eocene deposits of the Crimea Peninsula have been studied. They are assigned to the species *N. distans*. To compare the calcite crystallographic texture, we used shells of the bivalve mollusk *Placuna placenta* Linnaeus, 1758, collected on the Tamky City beach (Vietnam, coast of the South China Sea). Since the natural flat surface of the Nummulite test was measured, the valve of the bivalve *P. placenta*, which also has a natural flat surface, was chosen for comparison with the shell of a multicellular organism. It would be impossible to compare polished and unpolished objects. The inner surface of the *P. placenta* valve was measured. Besides, the shells of bivalve mollusks are very good for comparison, since world science has accumulated the most information about the crystallographic texture of minerals in the shells of these mollusks.

**Figure 3:**
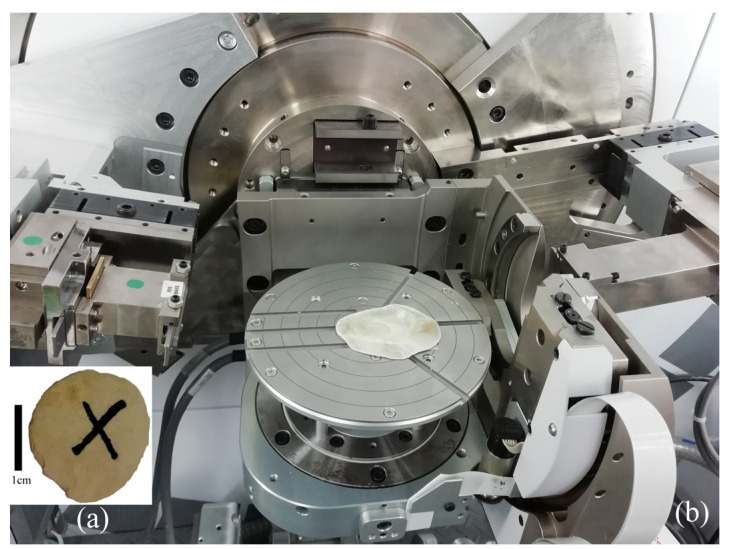
Studied samples of (**a**) nummulite and (**b**) *Placuna placenta* shell (Linnaeus, 1758) at the instrument Empyrean (Malvern PANalytical).

The study was carried out using X-ray diffraction (with Cu Kα radiation) at the instrument Empyrean (Malvern PANalytical) in the Frank Laboratory of Neutron Physics, Joint Institute for Nuclear Research (Dubna, Moscow region, Russia) (Figure 3).

The measurements were performed on bulk samples with a plane surface in reflection geometry. Prior to the texture measurement, X-ray phase analysis was conducted using the HighScorePlus, version 4.1 software package (PANalytical) and the PDF-2 database. X-ray scans for phase analysis were carried out with the following parameters: an X-ray tube with Ni filtered Cu Kα radiation (λ = 1.54 Å), a voltage of 40 kV and a current of 40 mA, and an exposure time of 300 s per frame.

The X-ray texture experiment is the most common type of texture experiment and is used for mass texture studies [19,20]. Pole figure measurements with X-rays are based on diffraction that occurs when X-rays are scattered by crystal lattice atoms. In this case, scattering arises as a result of forced oscillations of the electrons of atoms under the action of the electromagnetic field of the incident X-ray wave. This experiment is a surface method since the penetration depth of X-rays is relatively small. Depending on the radiation wavelength and material, the thickness of the half-attenuation layer has characteristic values ranging from units to tens of microns. The X-ray experiment is carried out as follows: First, the scattering angles are fixed, at which Bragg reflection from different crystallographic planes occurs. The sample is placed in the center of the goniometer circumference. The primary beam is incident on the sample at an angle *θ*, the reflected beam can be observed at an angle of *2θ* with respect to the incident beam if the focusing circle intersects the goniometer circle at the collimator and receiving slit positions and simultaneously touches the sample surface. When measuring the PF of a crystallographic plane (*hkl*), the angle *2θ* takes on a value in accordance with the Bragg law for the reflection from this plane and is kept constant. The spatial orientation of the sample in the reflection mode is changed by rotating the sample about two mutually perpendicular axes. The intensity of the reflected beam is measured at each position. We note two problems of the X-ray texture experiment. The tilt of the sample relative to the incident beam leads to a change in the area of the irradiated surface and the relative path of the incident and reflected beams in the sample. It leads to a change in the recorded intensity of the reflected beam. Therefore, absorption corrections are made to the measured intensity for different tilt angles. At small angles of beam incidence on the sample surface in the reflection mode, the focusing condition is violated; the defocusing effect appears to manifest itself as an additional drop in intensity with increasing tilt angle, and the correction for absorption does not provide a complete correction. Therefore, intensity corrections for defocusing are introduced, which are determined from measurements of reference textureless samples or using calculations. In addition, the defocusing effect leads to a broadening of the diffraction lines, and this broadening increases with an increase in the sample tilt. Starting from angles of χ~70°, the corrections become insufficient. Therefore, in a standard X-ray texture experiment, only incomplete PFs (up to 70–80°) can be obtained.

Pole figures were measured for the calcite crystallographic planes (0006) and (10–14) up to χ = 70° with step 5°. These diffraction reflexes were selected for measuring because they are the most intensive and have a pronounced signal/background ratio. Besides, it should be noted that two complete pole figures with Miller indexes (0006) and (10–14) are enough for calcite trigonal crystal symmetry to provide a comprehensive texture description. It yielded a total measuring time of approximately 4 h per pole figure at counting times of about 10 s per point. Complete pole figures were calculated using the orientation distribution function (ODF) reconstructed from the measured ones by the WIMF method [44]. ODF is a three-dimensional probability density function, which is the relative volume of grains with a particular orientation. It can be calculated using measured pole figures. ODF contains complete information about crystallographic texture and can therefore be used to recalculate complete pole figures from incomplete X-ray-measured ones. In addition, ODF can be used to calculate pole figures that cannot be measured.

Complete pole figures can be normalized, which is not possible for incomplete ones. Then the normalized pole density intensities (for example, maxima) can be compared with the corresponding values of pole figures measured for other samples. Let us explain the pole figure normalization. Let $\vec{y}$(χ,η) be the unit vector of a direction in the “sample coordinate system” [χ,η are spherical coordinates of the vector], $\vec{h}$(ϑ,φ) the unit vector of a direction described in the “crystal coordinate system” of a single crystal [ϑ,φ are spherical coordinates of the vector], then pole figure function $P_{\vec{h}}(\vec{y})$ gives the volume fraction of the sample for which the lattice plane normal $\vec{h}$ falls in various sample directions $\vec{y}$ [45]. By using the integration over sphere

(1) $$\int_{S^{2}}d\vec{y}=\int_{0}^{\pi }\sin\theta d\theta \int_{0}^{2\pi }d\phi =4\pi$$

one can write for the isotropic distribution:

(2) $$\int_{S^{2}}P_{\vec{h}}^{is}(\vec{y})d\vec{y}=4\pi .$$

Then, for an arbitrary distribution, we will have:

(3) $$\int_{S^{2}}P_{\vec{h}}(\vec{y})d\vec{y}=A.$$

If we introduce normalized pole figure:

(4) $$P_{{}_{\vec{h}}}^{norm}(\vec{y})=\frac{4\pi }{A}P_{\vec{h}}(\vec{y})$$

then

(5) $$\int_{S^{2}}P_{{}_{\vec{h}}}^{norm}(\vec{y})d\vec{y}=4\pi .$$

PANalytical X’pertTexture software, version 1.3 was used for calculation of ODF and complete pole figures [46]. Pole figures are normalized for a comparison of different samples. They are displayed in stereographic projection.

Only the outer wall of the samples was studied since the penetrating power of X-rays is low. A smooth surface was chosen on the nummulite test without additional grinding, which could distort crystallographic texture features in the least way. Measuring an altered surface could produce artifacts and distort the mineral texture.

In addition, the internal structure of the samples was studied in order to detect calcium carbonate abiogenic crystals grown inside or carbonate rock that got there, which would affect the results. The study was carried out with a Neoscan N80 X-ray microtomography, Belgium, Mechelen (Borissiak Paleontological Institute, Russian Academy of Science, PIN RAS, Moscow, Russia). The investigation parameters are the following: current 43 mA, voltage 92 kV, resolution 18.69 µm, rotation step 0.4°, Cu filter 0.25, and rotation by 180°.

The microstructure was studied using a Tescan/Vega2 scanning electron microscope, Czech Republic, Brno (PIN RAS) with gold sputtering on a chip and on a polished surface etched with a 10% hydrochloric acid solution.

The calcite crystallographic texture of *P. placenta* was also analyzed by X-ray diffraction on the inner, untreated, smooth shell surface.

## 3. Results

**Figure 4:**
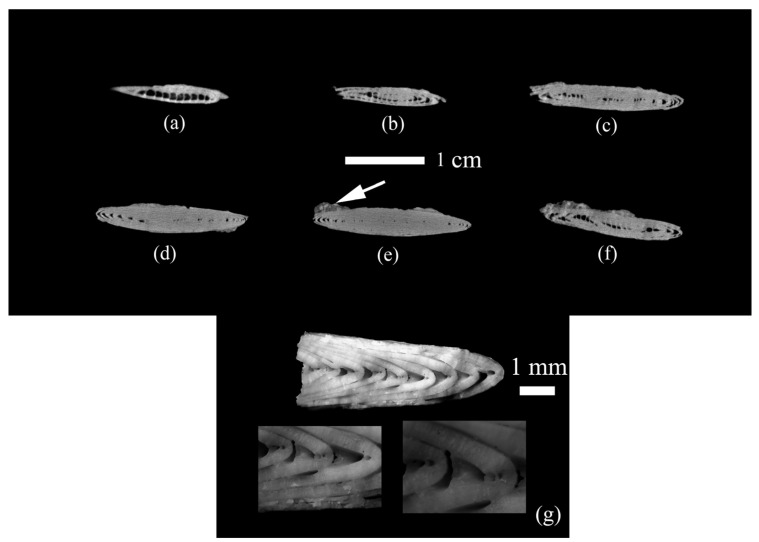
Internal structure of the nummulite sample: (**a**–**f**) virtual microtomographic sections, (**g**) test chips. The white arrow shows the host rock found only on the test surface.

**Figure 5:**
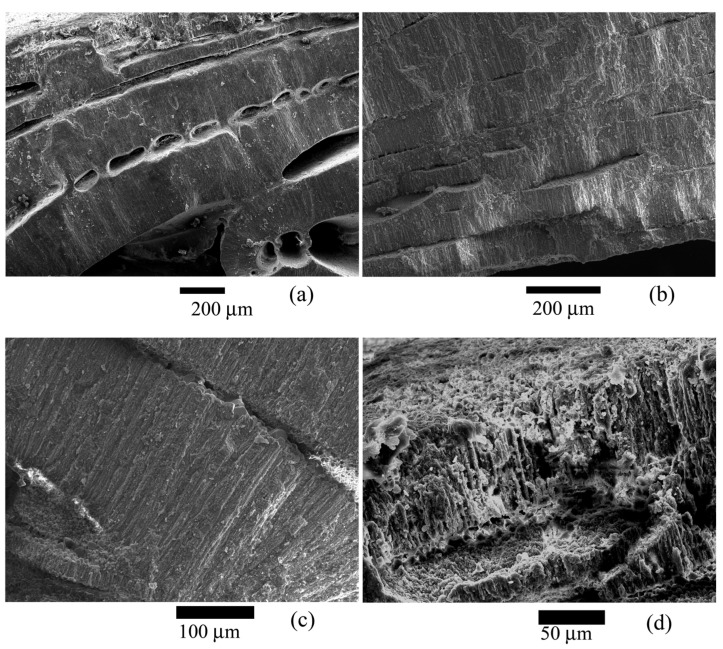
Microstructure of the studied *Nummulites distans* Deshayes, 1838 test. General picture. (**a**) Outer and inner walls of the *Nummulites distans* test with small unfilled voids; (**b**) Densely packed test layers; (**c**) Structure of the test wall. Densely arranged lamellae are visible. Their shape is not changed by recrystallization; (**d**) Destroyed outer layer of the test.

It was proved using X-ray microtomography that the space inside the test is not filled with calcite crystals of abiogenic origin or host rock. The test whorls are tightly adjacent to each other and to the outer test wall. The same was confirmed by the study on digital and scanning electron microscopes (Figure 4 and Figure 5).

**Figure 6:**
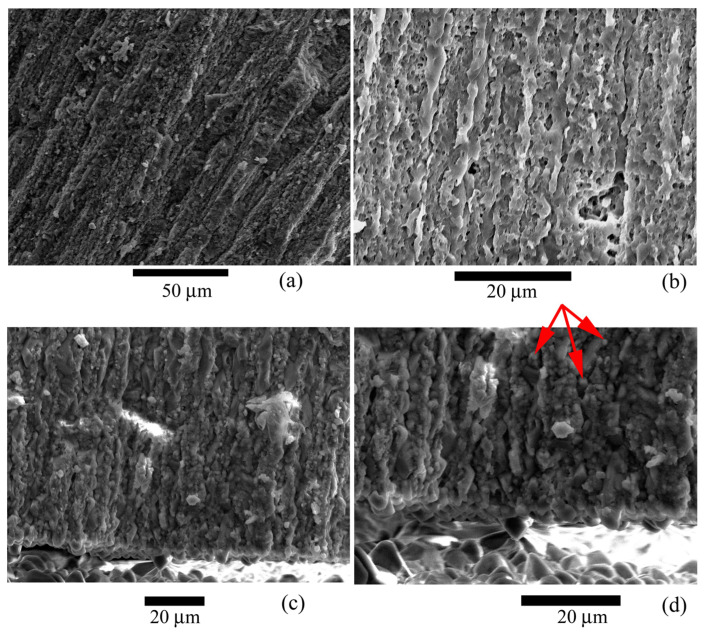
Microstructure of the studied *Nummulites distans* Deshayes, 1838 test: (**a**–**c**) Features of the shape and structure of the lamellae; (**d**) Structure of lamellae. Individual calcite crystals that make up the lamellae are visible. Large crystals are also visible at the border of the test walls, in the voids. Red arrows indicate individual crystals.

**Figure 7:**
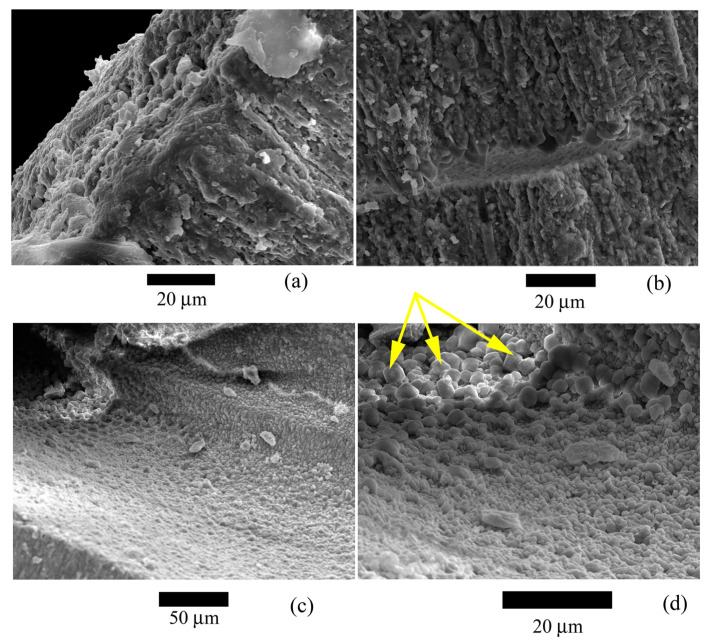
Microstructure of the studied *Nummulites distans* Deshayes, 1838 test: (**a**,**b**) Structure of lamellae and large crystals on outer (**a**) and inner (**c**,**d**) test surfaces. It can be seen on (**c**,**d**) how the crystals form polyhedra, probably around the pores. Large crystals (marked with yellow arrows) are probably not associated with test microstructure. They were formed posthumously.

It was found that the test wall is not homogeneous and consists of microstructural elements (Figure 5, Figure 6 and Figure 7), according to the electron microscopy results. These elements are narrow lamellae running perpendicular to the surface. They are located close to each other. The test wall is composed of hyaline [4]. Similar microstructural elements were observed in the species of the genus *Nummulites* Lamarck, 1801 [47] and in other Nummulitidae, for example, the genera *Spiroclypeus* Douvillé, 1905, and *Operculina* d`Orbigny, 1826, from Upper Oligocene deposits [48]. We have no reason to assume that the microstructures have undergone significant post-mortem changes and recrystallization since they are very similar to those depicted in ([49], Figure 1f; [48], plate 1.1, Figures 1 and 5; [50], Figure 4; [47], Figure 3d]). Besides, the test walls of recent foraminifera *Pulleniatina obliquiloculata* (Parker et Jones, 1865) also contain similar microstructural elements [39] found in the test wall of *N. distans*. A team of authors [40] proposed various possible postmortem changes in the mineral substance of the test wall. Of the four options for postmortem changes in tests (Ref. [40], Figure 5], only additional overgrowth of calcite crystals can be applied to our sample. Large calcite crystals are located on the boundary surfaces of the internal test structures; they probably belong to overgrown calcite crystals. Or it is postmortem marine cement, as has been noted for recent foraminifera and Eocene nummulites of North Africa [47]. These crystals are arranged in a single layer, and their number is insignificant compared with the crystals in the test walls. Any possible abiogenic calcite crystals inside the test are contained in negligibly small quantities and cannot significantly affect the pole figure. If recrystallization were on a large scale, it would affect the intensity of the crystallographic texture, and it would not be so sharp.

Microstructural elements are not single crystals; the image clearly shows that the lamellae consist of numerous crystals (Figure 6c,d and Figure 7b). Large crystals are located on the test surface (Figure 7a). Similar crystals were found on the inner surfaces of the test (Figure 7b–d). The penetrating power of X-rays is up to 200 µm, which approximately corresponds to the outer wall of the nummulite test (Figure 5). That is, only this part of the wall was measured, and no internal structures were studied.

Therefore, the orientation of multidirectional test structures (for example, vertical) did not affect the variety of crystal orientations. The microstructure and the general structure of the test were studied only with the aim of proving the polycrystalline nature of the lamellae of nummulite tests, the absence of recrystallization, and the host rock. However, it is impossible to directly relate microstructure and crystallographic texture. This was shown in the work [51].

**Figure 8:**
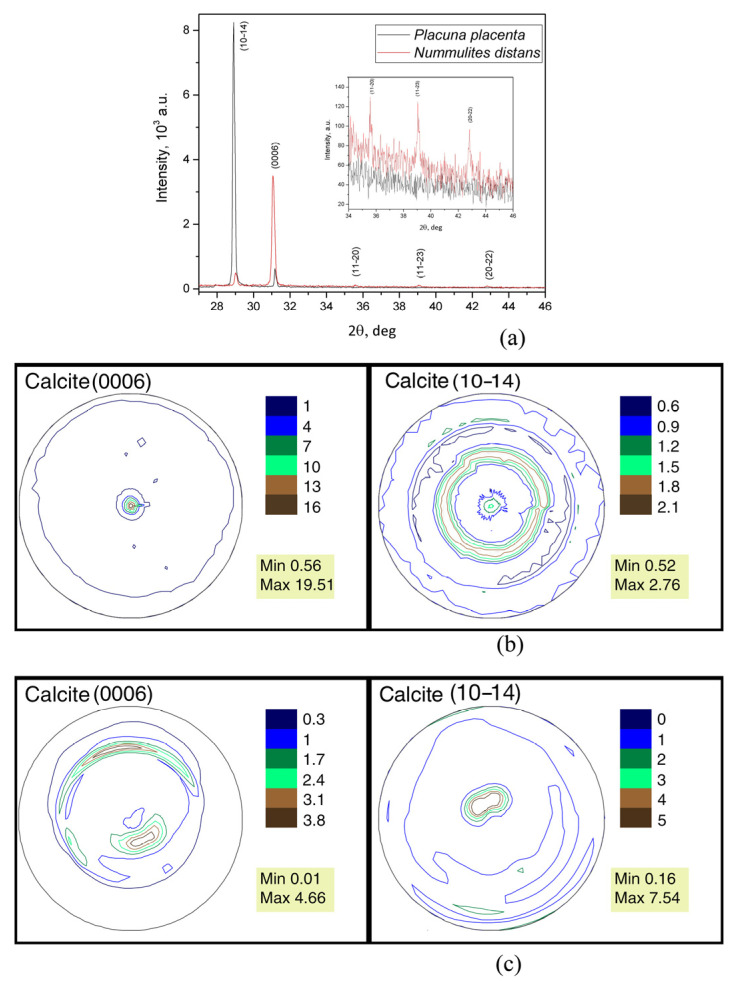
X-ray diffraction results: (**a**) X-ray patterns with indexed calcite reflections for *Nummulites distans* Deshayes, 1838 test and *Placuna placenta* Linnaeus, 1758 shell. The inset shows the X-ray patterns of calcite for *Nummulites distans* test and *Placuna placenta* shell with enlarged scale. (**b**) Calcite pole figures of the *Nummulites distans* Deshayes, 1838 test; (**c**) Calcite pole figures of the mollusk *Placuna placenta* Linnaeus, 1758 shell.

It can be concluded that the nummulite test consists only of calcite based on the X-ray diffraction results. It has trigonal crystal symmetry with space group R-3c (No. 167, the card with the reference code 01-076-2712, is chosen for calcite from the PDF-2 data base [52]). X-ray patterns with indexed calcite reflections for the *Nummulites distans* test and *Placuna placenta* shell are presented in Figure 8a. This figure clearly shows the difference in the relative intensity of the corresponding reflections in the diffraction patterns for the two samples. The inset is presented with an enlarged scale in Figure 8a to show the difference between the reflections with small intensities.

The isoline pattern clearly shows that the (0006) pole figure is axial.

The center of the pole density maximum is one; it coincides with the pole figure center. The isolines pass close to each other near the pole figure center (Figure 8). The calcite pole density maximum of (0006) pole figure for the nummulite test that can be interpreted as crystallographic texture sharpness turned out to be surprisingly very large, 19.51 mrd. The (10–14) pole figure is also axial but differs in the isoline pattern. There are two centers of the pole density maxima (Figure 8b), and they are unequal. One of them is located in the pole figure center, but its sharpness is less than that of the second. The second maximum encloses the first one in the form of an open ring. That is, the crystals have more dominant directions than are observed in the (0006) pole figure. This is reflected by the values of the pole density maxima.

The sharpest calcite crystallographic texture was noted in the fossil shells of *Mytilus galloprovincialis* Lamarck, 1819, from the Karangat deposits (Upper Pleistocene) of the Taman Peninsula, Tuzla Spit. The calcite crystallographic texture sharpness is 15.72 mrd [30]. These data were obtained using neutron diffraction. The pole figures in both cases are normalized, and therefore they can be compared with each other. The texture sharpness of the (10–14) calcite pole figure of the nummulite test is many times less, only 2.76 mrd. This is close to the value of the highest sharpness of the Pleistocene mussel, 3.31 mrd. The lower intensity of the calcite pole density at the {10–14} pole figure of the *N. distans* test is associated with the multiplicity factor of the atomic planes of the crystal lattice, which are equivalent in terms of symmetry.

Calcite crystals in bivalves are formed by epithelial cells of a special organ, the mantle [53]. Their synthesis occurs outside of the tissue cells. It was revealed that the isolines on the pole figure (0006) of the *P. placenta* are less compactly distributed when comparing the calcite crystallographic texture of the mollusk, also measured using X-ray diffraction, with the calcite texture of *N. distans* foraminifera. Besides, there are two texture sharpness centers of crescent and oval shape, and the pole density maximum on the pole figure (0006) of the *P. placenta* is 4.66 mrd (Figure 8c). This is a rather high value, but several times inferior to that of foraminifer.

The calcite pole figure (10–14) has a more compact isoline pattern; the center of pole density maximum is one, and it is located close to the center. The pole figure density maximum is 7.54 mrd. This value is high, and we did not find it for any of the studied objects that had calcite shells. This fact requires separate consideration. The pole density maximum of this pole figure is almost three times higher than the value of nummulite.

Interestingly, the previously measured pole figures of *P. placenta* using the EBSD method [54,55] do not coincide with ours. This can happen because EBSD is more local than the X-ray method. Therefore, it is difficult to compare the results obtained by these different approaches.

## 4. Discussion

It is amazing that a unicellular organism is able to create a complex test with multiple whorls and chambers. Even more surprisingly, the crystalline organization of calcite in these tests is highly ordered, even more so than in metazoa (metazoans) such as bivalves. Many works are devoted to the biological control by the organic matrix of the growth of crystals and microstructural elements and the impact of various biogenic substances on this growth [56,57,58,59,60].

Of course, here we can only talk about matrix biomineralization [1]. That is, the level of biomineralization is very high already in unicellular organisms. They are characterized by a large ordering of crystal orientations in the test. The first secretory calcite foraminiferal tests appeared in the Cambrian in the order Parathuramminida; they were microgranular [17]. Secretion tests were most developed in the Devonian [43], but they were mostly microgranular. Hyaline tests, which are also characteristic of nummulites, appeared at the end of the Permian in the family Nodosariidae [43]. Foraminifers with a similar test wall were common in the Mesozoic and Cenozoic. This structural feature became one of the key ones for identifying the suborder Rotaliina, which previously included the family Nummulitidae [42].

The study of the crystallographic texture of the foraminiferal test allows us to answer the question of how ordered the crystalline orientations of its generatrixes are. One can understand when such a test could appear in the case of strict crystalline ordering. The degree of crystal ordering can be described by the crystallographic texture, which is a set of preferred crystallographic orientations of the constituent crystalline grains [18]. That is, a test with strictly ordered crystal orientations could appear at the border of two eras: the Paleozoic and the Mesozoic. It is from this time that we can point to the appearance of foraminiferal tests, which are characterized by a high order of calcite crystals. Unfortunately, the tests of early representatives are very small, and their surface is too uneven to allow X-ray measurements because we studied the objects with an undisturbed natural flat surface that were suitable in size for the equipment used.

It is important to understand the process of organic matrix control over the arrangement of calcite crystals that occurs in foraminifera in order to reproduce it artificially for the production of materials with high crystal ordering without the use of energy-intensive technologies, such as high temperatures and pressures [61].

## 5. Conclusions

As a result, it was revealed that unicellular protozoa—foraminifera *N. distans*—are able to build a test with a high degree of calcite crystal ordering. It means that strict control over the growth of crystals occurs already at the cell level. The calcite crystallographic texture and sharpness of the bivalve mollusk shell are much lower than in the foraminiferal test. In this case, the mollusk shell is built from a specialized fabric of the mantle. Understanding the processes of very sharp crystallographic texture formation may be useful for designing artificial materials with predefined properties.

## Data Availability

The data supporting reported results can be obtained on request from the article authors.
